# Tight Rings

**Published:** 1881-06

**Authors:** 


### TIGHT RINGS.

Treatises on operative surgery are absolutely silent on the constriction, by rings, of fingers swollen from one cause or another, and on the method of removing them. The accident is, nevertheless, of common occurrence, causes great pain, sometimes gives rise to great uneasiness, and may even threaten the safety of the finger itself. As a rule, in these cases of constriction, the ring is cut unnecessarily, for want of a simple method of removing it, notwithstanding the popular plan, which comes to us by tradition, and is thus described by Oribasius, vol. iv, p. 251, Daremberg's edition. He writes: "Sometimes the finger is constricted by a ring; and it is necessary to remove the ring without delay, by giving it a rotatory motion; bathing at the same time the finger with warm water, and greasing it with some kind of fatty matter. If the ring does not yield to these efforts, the following operation is recommended: A thick and twisted thread is sharpened at one end in the same way as cobblers sharpen their threads, and passed between the finger and the ring, whilst the rest of the thread is rolled round the finger. When this thread is unrolled, the ring moves towards the tip of the finger, whence it can be removed. If the ring resists this treatment, it is then necessary to cut it." Aetius, who lived at the end of the fifth and the beginning of the sixth centuries, repeats the recommendations of Oribasius. A writer in the *Concours Medical* suggests some improvements on the plan, so as to reduce the volume of the finger by ischæmiatising it, in the same way as ischæmia is produced with Esmarch's bandage. In the first place, the finger is coated with fatty matter; then a thin thread, about a yard and a quarter long, is taken; one end is placed under the ring, and passed above it with a pair of pincers to the length of about three inches. The end of the thread being thus fixed by the ring, the rest of the thread is taken to the top of the finger, round which it is rolled in close overlapping lines, not leaving any space between them. This done, the second end of the thread is also passed under, and brought up above the ring. Then, this end being taken between the fingers, the rest of the thread is unrolled resting on the ring, which is thus gradually brought up to the point, where it is easily removed. If a first trial does not always succeed, it is rare for the ring not to yield to efforts twice or thrice repeated. Should this be the case, the ring, of course, must be cut on a cannulated sound with a file or divider.—*Clinical News.*

